# Quality and Reliability of YouTube Videos on Myocardial Infarction: A Cross-Sectional Study

**DOI:** 10.7759/cureus.43268

**Published:** 2023-08-10

**Authors:** Shubham Holge, Amaresh Gogikar, Rafiya Sultana, Urvashi Rathod, Chandramouli Chetarajupalli, Yarrabathina Laxmi Supriya

**Affiliations:** 1 Community Medicine, Dr. Shankarrao Chavan Government Medical College, Nanded, IND; 2 Internal Medicine, Rangaraya Medical College, Kakinada, IND; 3 Internal Medicine, Nimra Institute of Medical Sciences, Vijayawada, IND; 4 Internal Medicine, Narendra Modi Medical College, Ahmedabad, IND; 5 Internal Medicine, Guntur Medical College, Guntur, IND; 6 Internal Medicine, Gandhi Medical College, Secunderabad, IND

**Keywords:** video power index, discern score, global quality score, youtube, heart attack

## Abstract

Introduction: This study aims to assess the quality and reliability of the disease information available on YouTube (www.youtube.com) about “heart attacks” or myocardial infarctions, using a Global Quality Score (GQS) for quality, a DISCERN score for reliability, and a Video Power Index (VPI) for popularity.

Methodology: In this cross-sectional observational study, the YouTube videos were analyzed in terms of the type of uploader, content, and other factors. The GQS, DISCERN score, and Video Power Index (VPI) were utilized to assess the quality, reliability, and popularity of the information, respectively.

Results: The majority of the videos (78.44%) were uploaded over a year ago. Only 33.34% and 7.84% were uploaded by doctors and healthcare organizations, respectively. Around 72.55% of the videos contained information about symptoms, 66.67% discussed the causes, 52.94% covered treatment, and 47.06% focused on prevention. Additionally, 41.18% provided details on investigations, while only 19.61% touched upon mortality. Patient-created videos accounted for 19.61% of the content, and 15.69% of the videos included promotional material.

Conclusion: The main outcome of our study indicates that the YouTube videos examined regarding myocardial infarctions exhibit high-quality content, as supported by a higher average GQS score. The consistent quality of information discovered in our study suggests that YouTube can serve as an additional platform for sharing knowledge and educating individuals about this important health condition. By raising awareness and delivering accurate information, these videos can help in early detection, prevention, and better outcomes for individuals who are at risk of experiencing a myocardial infarction.

## Introduction

Heart attack is a cardiovascular disease and is one of the most common causes of death worldwide [1]. Heart attacks, also known as myocardial infarctions, occur when a part of the heart muscle does not receive sufficient blood flow [2]. Early diagnosis of this condition is crucial for effective treatment and to avoid death overall [1]. Major risk factors predisposing to heart attacks include hypertension, obesity, smoking, and high blood cholesterol levels. Commonly reported symptoms are radiating pain in the shoulders, dizziness, chest discomfort, and breathlessness [2].

YouTube (www.youtube.com) has become one of the most popular resources for gaining information nowadays. More than 2 billion people watch content on this online platform daily for a minimum of 15 minutes. People with chronic ailments depend largely on information obtained from social media, YouTube being one of them. YouTube shows great potential to act as a medium for relaying any health-related information to the common man [3].

There is an expanding body of research assessing the accuracy of healthcare information on this platform including conditions like chronic obstructive pulmonary disease (COPD), abdominal aortic aneurysms (AAA), narcolepsy, and many others [4-10]. Since heart attack is one of the major chronic illnesses prevailing in today’s population, and with the increasing usage of social media for health-related concerns, it is of utmost importance to ensure that accurate information pertaining exclusively to heart attacks is being shared online. This study aims to assess the quality of the information related to heart attacks available to patients on YouTube. To facilitate the distribution of reliable information related to heart attacks to the general public, it explores the consequences of these findings and offers recommendations for future research.

## Materials and methods

This cross-sectional observational study was conducted in April 2023, with data collection completed on April 03, 2023. A structured questionnaire was developed using Google Forms to collect information on predetermined criteria related to videos including duration of videos, number of likes, dislikes, views, and comments. The content of the videos was assessed in terms of information related to symptoms, etiology, treatments, prevention, investigation, mortality, rehabilitation, support groups, personal experience shared by individuals or parents, and promotional content by pharmaceutical companies or doctors. 

Additionally, Global Quality Scores (GQS), DISCERN score, and Video Power Index (VPI) where the popularity of the video is evaluated based on likes and dislikes were assessed.

The GQS is a rating system with five grades that evaluates the overall quality of health-related websites. It offers a general evaluation of website quality without specifying particular aspects. A total score of 4-5 indicates high quality, 3 indicates moderate whereas 1-2 indicates low quality. 

The DISCERN tool is used to assess the reliability of YouTube videos. The scale consists of five yes/no questions. The answer to each question as “yes” is scored 1 and “no” as 0, the total score is then calculated. The total score ranges from 1 (lowest) to 5 (highest), with a higher score indicating a higher level of educational quality [11]. Additionally, we calculated two metrics to assess the popularity of the videos: the "like ratio" (calculated as the number of likes divided by the sum of likes and dislikes, multiplied by 100) and the "video power index" (VPI), which is determined by multiplying the like ratio with the view ratio divided by 100. These metrics, namely the like ratio and VPI, were utilized to gauge the popularity of the videos [12]. These responses were recorded in Google Sheets and transferred to Microsoft Excel for further analysis.

The videos were searched using specific keywords related to heart attacks, including “heart attack”, “myocardial infarction”, “heart attack treatment”, “heart attack prevention”, “heart attack cause”, and “heart attack exercise”.

A total of 51 videos that fulfilled the predetermined inclusion criteria were collectively assessed by the authors. These criteria required the videos to be relevant to the topic of heart attacks, presented in English, and have a duration of 1 to 20 minutes. Videos that did not meet these criteria were excluded from the study. Before conducting the statistical analysis using SPSS software (IBM Corp., Armonk, NY), any duplicate entries were removed from the dataset.

## Results

After following inclusion-exclusion criteria and deletion of duplicates, 51 videos were selected for this study. 

The characteristics of the YouTube videos analyzed are presented in Table 1. The videos analyzed had a widespread reach of 74,898,733 viewers. Most of the videos (78.44%) were uploaded more than a year ago and only 33.34% and 7.84% were uploaded by doctors and healthcare organizations. 

**Table 1:** Characteristics of YouTube Videos Analyzed

Characteristics of YouTube videos	Number (Percentage)
Time since uploaded	
More than a month to six months (31 - 180 days old)	04 (7.84%)
More than six months to last one year (180 - 365 days)	07 (13.72%)
More than one year (> 365 days)	40 (78.44%)
Popularity	
Total no. of views	74898733
Total no. of likes	554348
Total no. of dislikes	33166
Total no. of comments	16128
Type of uploader	
Doctor	17 (33.34%)
Hospital	10 (19.6%)
Healthcare organization	04 (7.84%)
News channel	04 (7.84%)
Other	16 (31.38%)

Table 2 presents the type of information about heart attacks conveyed by these videos. Around 72.55% of the videos provided information on symptoms, 66.67% on cause, 52.94% on treatment, 15.69% on rehabilitation and promotional content, and 3.92% on support groups.

**Table 2:** Information Provided by YouTube Videos Regarding Heart Attacks

Type of Information Provided by the YouTube Videos	Number of Videos (Percentage)
Description of symptoms	37 (72.55%)
Information about cause/etiology	34 (66.67%)
Info about investigations/tests	21 (41.18%)
Info about prevention/vaccines	24 (47.06%)
Info about treatment	27 (52.94%)
Info about mortality	10 (19.61%)
Info about rehabilitation	8 (15.69%)
Info about support groups	2 (3.92%)
Info about people/patients sharing their own experience	10 (19.61%)
The post has promotional content by pharmaceutical companies or by doctors	8 (15.69%)

Table 3 shows the comparison of the quality and reliability of information about heart attacks on YouTube between the different types of uploaders. The mean GQS of doctors, news agencies, and others is 4, and hospitals and healthcare organizations have a score of 3.5 and 2.5 respectively. The mean VPI of news agencies is the highest at 758.93 compared to healthcare organizations at 345.65, others at 221.90, hospitals at 201.85, and least for doctors at 154.80. The DISCERN Score is 4 for doctors, 3.5 for news agencies and others, and 3 for hospitals and healthcare organizations. 

**Table 3:** Comparison of Global Quality Scores (GQS), DISCERN Scores, and Video Power Index (VPI) Based on the Type of Uploader

Assessment Tools	Doctors (n=17)	Hospitals (n=10)	Healthcare organizations (n=4)	News agencies (n=4)	Others (n=16)	P-value (0.05) (Kruskal-Wallis test)
	Median (IQ1, IQ3)	Median (IQ1, IQ3)	Median (IQ1, IQ3)	Median (IQ1, IQ3)	Median (IQ1, IQ3)	
VPI	154.80 (42.02, 304.93)	201.85 (40.84, 815.27)	345.65 (27.31, 624.99)	758.93 (4.90, 12454.43)	221.90 (37.68, 1846.56)	0.937
GQS	4 (3.5, 4.5)	3.5 (3, 4)	2.5 (2, 4.5)	4 (3.25, 4.75)	4 (3, 4)	0.422
DISCERN Score	4 (3, 4)	3 (3, 4)	3 (1.5, 3.75)	3.5 (2.25, 4)	3.5 (3, 4)	0.592

Data values were analyzed using the Kruskal-Wallis test. However, the difference in the mean VPI, GQS, and DISCERN Score value is statistically insignificant among all uploaders.

## Discussion

Although there has been significant advancement in the treatment of acute myocardial infarction (AMI) or coronary artery disease, it continues to be one of the leading causes of death worldwide. AMI claims the lives of more than a million Americans annually and affects millions of people globally [13]. Raising awareness about myocardial infarction on a global scale is crucial due to its significant impact on society. With the increasing use of smartphones, YouTube has become a popular platform for video sharing and a valuable resource for both the general public and healthcare professionals [14]. Therefore, evaluating the relevance, reliability, and quality of YouTube videos related to myocardial infarction is imperative.

Previous studies have shown the effectiveness of video-based information in enhancing understanding of various aspects of diseases, such as causes, signs and symptoms, diagnostic tests, prevention, and treatment options [14]. In our research, we evaluated the quality of videos based on factors like popularity, duration, source, content, and additional metrics provided by the source, such as the GQS, DISCERN score, and VPI.

In our analysis of 51 videos, the majority of the content focused on highlighting symptoms, causes, and etiology of myocardial infarction. These findings are consistent with our study's focus and are similar to the study conducted by Pant et al., which reported similar results (58%) regarding the emphasis on symptoms and etiology [14]. Additionally, we found that only a small percentage (3.92%) of the videos addressed the importance of support groups.

It is worth noting that although most videos were over a year old, they still received substantial viewership on YouTube. The uploaders of these videos varied, with doctors contributing significantly (33.34%), followed by educational channels (31.38%). Hospitals, healthcare organizations, and news outlets also provided video content in varying proportions (19.6%, 7.84%, and 7.84%, respectively).

Furthermore, our analysis revealed that a higher percentage (47.06%) of the videos in our study focused on prevention, which significantly differs from the findings of Pant et al.'s study which reported a lower percentage (17%) covering this topic [14]. Unlike their study, which included a larger proportion of videos from the past year (64%), our study included 21.56% of videos uploaded within the last year. Additionally, we established specific inclusion criteria, requiring the video length to be between 2 and 20 minutes.

In this study, we introduced a novel approach by utilizing the GQS, DISCERN score, and VPI as tools to assess the quality, reliability, and popularity of video content from each uploader. Fialho et al. conducted a study evaluating 1000 videos, excluding non-Portuguese content, and utilized different quality evaluation systems like the Health on the Net Code in addition to DISCERN scores to determine information accuracy [15]. A systematic review by Osman et al. in 2019 analyzed 202 English-language articles on medical and health-related content and found that 25 studies used the GQS system, with an average score of 2.68 out of 5 for all the videos in the study. However, in our study, the average GQS score was 3.6, indicating higher quality compared to previous research [16].

Despite doctors' lack of confidence in recommending YouTube as a reliable source of information, as supported by the study by Szmuda et al., regarding content for stroke, our study revealed consistent quality in the content related to myocardial infarction on YouTube [17]. We found no significant difference in the mean values of VPI and DISCERN scores, indicating the reliability and consistent quality of information on YouTube for myocardial infarction.

Limitations

This research, conducted as a cross-sectional study, focused on a subset of the information available on YouTube. Specifically, 51 videos were examined to gather data. To ensure a reliable sample, the videos included in the study were within the 2-20 minute range, as research indicates that the majority of YouTube users do not watch videos longer than 20 minutes. YouTube's vast content volume is evident, with 48 hours of video being uploaded every minute, resulting in a significant amount of content added daily. It is important to acknowledge that the data collection and evaluation occurred until April, and since then, there may have been notable changes in the available information on the platform that could potentially affect the GQS and VPI scores. Moreover, it is worth noting that video optimization and analytics can influence the search results and ranking of the same search item for different users.

## Conclusions

Our study's primary finding reveals that the YouTube videos analyzed concerning myocardial infarction demonstrate good quality content, supported by a higher average GQS.

The implications of our findings emphasize the necessity for reputable health organizations, such as the American Heart Association or the American College of Cardiology, to play a more active role in producing myocardial infarction-related videos. While these organizations were absent from the analyzed videos, their valuable expertise and authoritative content could significantly contribute to raising awareness about this leading cause of death. Including videos from these organizations would enhance the reliability and credibility of the information available on YouTube.

Future studies can contribute to the existing literature by exploring alternative keywords or search criteria. Analyzing different keywords could provide a more comprehensive understanding of the video landscape and potentially uncover additional gaps in information coverage. Furthermore, focusing on specific aspects such as prevention, treatment options, or patient experiences may offer unique insights and complement existing research.

Our study's practical implications suggest that healthcare professionals and the general public can utilize YouTube as a valuable resource for accessing reliable and informative information on myocardial infarction. The consistent quality of the information found in our study indicates that YouTube can serve as a supplementary platform for disseminating knowledge and educating individuals about this critical health condition. By increasing awareness and providing accurate information, these videos can contribute to early recognition, prevention, and improved outcomes for individuals at risk of myocardial infarction. Additionally, our study emphasizes the importance of collaboration between reputable health organizations and content creators to ensure the availability of reliable and trustworthy information on YouTube.
